# Dimension Reduction for Hyperspectral Remote Sensor Data Based on Multi-Objective Particle Swarm Optimization Algorithm and Game Theory

**DOI:** 10.3390/s19061327

**Published:** 2019-03-16

**Authors:** Hongmin Gao, Yao Yang, Xiaoke Zhang, Chenming Li, Qi Yang, Yongchang Wang

**Affiliations:** 1 College of Computer and Information, Hohai University, Nanjing 211100, China; gaohongmin@hhu.edu.cn (H.G.); rcyyang@hhu.edu.cn (Y.Y.); 1606020202@hhu.edu.cn (Q.Y.); wangyongchang@hhu.edu.cn (Y.W.); 2 School of Public Administration, Hohai University, Nanjing 211100, China; xkzhang@hhu.edu.cn

**Keywords:** multi-objective particle swarm optimization, game theory, hyperspectral remote sensor data, dimension reduction, band selection

## Abstract

Information entropy and interclass separability are adopted as the evaluation criteria of dimension reduction for hyperspectral remote sensor data. However, it is rather single-faceted to simply use either information entropy or interclass separability as evaluation criteria, and will lead to a single-target problem. In this case, the chosen optimal band combination may be unfavorable for the improvement of follow-up classification accuracy. Thus, in this work, inter-band correlation is considered as the premise, and information entropy and interclass separability are synthesized as the evaluation criterion of dimension reduction. The multi-objective particle swarm optimization algorithm is easy to implement and characterized by rapid convergence. It is adopted to search for the optimal band combination. In addition, game theory is also introduced to dimension reduction to coordinate potential conflicts when both information entropy and interclass separability are used to search for the optimal band combination. Experimental results reveal that compared with the dimensionality reduction method, which only uses information entropy or Bhattacharyya distance as the evaluation criterion, and the method combining multiple criterions into one by weighting, the proposed method achieves global optimum more easily, and then obtains a better band combination and possess higher classification accuracy.

## 1. Introduction

With the development of high-resolution optical sensors, hyperspectral remote sensing images (HSIs) are achieved, which consist of hundreds of different spectral bands of the same remote sensing scene. A HSI is different from general multispectral remote sensing image. A HSI can express the two-dimensional spatial information of the Earth’s surface and increase its one-dimensional spectral information. Therefore, the entire HSI can be regarded as an “image cube.” As the band number increases, the magnitude of the hyperspectral data increases geometrically. The HSI has the characteristics of a high spectral resolution, large number of bands, large amount of data, and high information redundancy [1,2]. These characteristics pose considerable challenges to the storage, transmission, and processing of HSIs. Dimension reduction can effectively reduce the amount of HSIs, and then speed up the processing of hyperspectral sensor data. In addition, it can also extract important features of hyperspectral sensor data at the same time. Thus, dimension reduction is necessary before hyperspectral image processing is performed. It is of great significance to further promote the development of the hyperspectral sensor’s application topics, such as vegetable, agriculture, oil, geology, urban, land use, water resources, and disaster [3]. The dimension reduction method can be divided into the feature extraction method and feature selection method. The band selection method can reduce some unnecessary bands, which can reduce the computation cost of classification and effectively avoid the Hughes phenomenon [4,5]. However, commonly used feature extraction methods involve the transformation of an original feature space through linear or nonlinear transformation and its projection to a low-dimensional feature space [6]. These methods achieve the recombination and optimization of spectral bands. These methods include principal component analysis [7,8] and independent component analysis [9]. After projection is completed, the arrangement order of the original band and the correlation among bands change. Consequently, the physical characteristics of the spectrum are destroyed and spectral information is lost, thus affecting the improvement of the classification accuracy.

Band selection is the most commonly used method for the dimension reduction of hyperspectral images. Band selection mainly adopts information entropy or interclass separability as evaluation criteria. Ge et al. [10] used mutual information as evaluation criteria, clustered the bands, and selected the bands of each cluster center as the best band combination. Liu et al. [11] utilized the Kullback–Leibler divergence to express information and proposed an unsupervised band selection method. Padma et al. [12] used the Jeffries–Matusita (J–M) distance to select the optimal band combination. Huang et al. [13] proposed a hyperspectral band selection method based on particle swarm optimization (PSO) and sequential search in 2012. These band selection methods adopt single criterion to search for the best band combination, which is unilateral. When the information entropy is used as the evaluation criterion, the band combination with high information entropy is selected. However, the statistical distance between categories in the selected band combination may not be the largest; that is, the total interclass separability may not be the best. Similarly, when the interclass separability is used as the evaluation criterion, the information entropy of the selected band combination may not be the highest, but it may affect the classification accuracy. Gurram et al. [14] proposed a band selection method based on an alliance game. This method divides all bands into several band subsets and uses the Shapley value method in the alliance game theory (GT) to calculate the contribution of each band subset to classification. The band subset with a large contribution to classification can be selected by setting a threshold to participate in classification and achieve the dimension reduction of HSI. However, it requires to traverse all band subsets as much as possible, thereby causing high computation complexity.

Considering the one-sided nature of the band selection method with single evaluation criterion, some scholars proposed a band selection method based on multiple criteria (hereinafter referred to as the multi-criteria band selection method). Wang et al. [15] adopted the J–M distance and the best index factor as the evaluation criteria and combined the two evaluation criteria by using the weighting method. Gao et al. [16] used the Choquet fuzzy integral to combine information entropy, the correlation between bands, and interclass separability to select bands. Multi-criteria band selection can be considered a multi-objective optimization problem. Multiple evaluation criteria correspond to multiple targets to be optimized. Band selection aims to find the band combination that can optimize multiple targets. The basic strategy of these multi-criteria band selection methods is to convert multiple targets into a single target, and then use a single-target-based search algorithm to determine the optimal band combination.

However, a multi-objective optimization problem is different from a single objective optimization problem. It has a remarkable characteristic, that is, the various subgoals of the multi-objective optimization problem are conflicting. The improvement of one subgoal may cause the performance degradation of another subgoal or other subgoals. That is, making multiple subgoals achieve the optimal simultaneously is impossible. Each subgoal can be optimized by coordinating and compromising the subgoals. Lee et al. [17], Lee et al. [18], and Zamarripa et al. [19] proposed to combine GT with the multi-objective optimization problem and applied GT to solve optimization problems [20] with constraints, multiple goals, and different objective functions.

In this case, this work proposes to adopt the correlation between bands as a constraint and apply GT to optimize two targets, namely, information entropy and interclass separability. The optimal band combination is determined by constructing and combining a game model with multi-objective PSO. Finally, the experiments are designed to verify the effectiveness of the proposed method.

## 2. Multi-Objective PSO Algorithm and GT for Dimension Reduction of Hyperspectral Images

In this section, the multi-objective optimization problem, PSO algorithm, and GT are briefly introduced. Then, the multi evaluation criterion function of the proposed method is provided. Finally, the implementation steps of the proposed method are described in detail.

### 2.1. An Overview of Multi-Objective Optimization Problems

In reality, many research and engineering problems should simultaneously optimize multiple targets. However, many targets have mutual constraints. In the optimization of one target, other targets become affected and worsen because optimizing multiple targets is difficult. The solution of multi-objective optimization problems is often standard, but a compromise solution set, which is called a Pareto-optimal set or nondominated set, exists. The general representation of the multi-objective optimization problem is as follows:

A multi-objective optimization problem is assumed to be a maximization problem, which is composed of *N* decision variables, *m* objective functions, and *K* constraints:

(1) $$\left\{ \begin{array}{l} \mathrm{Maximize}\;y=F(x)=(f_{1}(x),f_{2}(x),\ldots ,f_{m}(x))\\ s.t.\;e(x)=(e_{1}(x),e_{2}(x),e_{3}(x),\ldots ,e_{k}(x))\leq 0\\ \mathrm{where}\;x=(x_{1},x_{2},x_{3},\ldots ,x_{n})\in X\\ \;y=(y_{1},y_{2},y_{3},\ldots ,y_{m})\in Y \end{array}\right.$$

where *x* is the decision vector, *y* is the target vector, *X* is the decision space, *Y* is the target space, and the constraint condition, *e*(*x*), is the range of the decision volume.

In the early stage, the multi-objective optimization problem is usually converted into a single objective optimization problem through the weighting method and solved through mathematical programming. However, objective and constraint functions may be nonlinear and discontinuous, and mathematical programming cannot achieve ideal results. The intelligent search algorithm has self-organizing, self-adaptive, and self-learning characteristics and is non-restricted by the nature of the problem. The intelligent search algorithm can be used to solve the multi-objective optimization problem, which is high dimensional, dynamic, and complex. In 1967, Rosenberg proposed to use the intelligent search algorithm to solve multi-objective optimization problems, but it was not implemented. After the birth of the genetic algorithm (GA), Schaffer proposed a vector evaluation GA, which was the first time that the intelligent search algorithm was combined with the multi-objective optimization problem. The intelligent search algorithm has been developed into the mainstream algorithm for multi-objective optimization problem solving. Moreover, a number of classical algorithms have been designed.

### 2.2. PSO Algorithm

PSO shows a simple concept, provides easy programming, and requires few parameter adjustments. Some scholars confirmed that the multi-objective PSO algorithm has obvious advantages in three aspects, namely, speed, convergence, and distribution [21,22].

A group with *n* particles is randomly initialized, assuming that the population is $X=\left(X_{1},X_{2},\cdots ,X_{n}\right)$ in a *D*-dimension search space. When particles fly at a certain speed in space, the position state property of particle *i* is set as follows: Vector: $X_{i}={\left(x_{i1},x_{i2},\cdots ,x_{1D}\right)}^{T}$. The fitness values corresponding to each particle position can be calculated based on the objective function. The speed vector of particle *i* is $V_{i}={\left(V_{i1},V_{i2},\cdots ,V_{1D}\right)}^{T}$, and the individual optimal position vector is $P_{i}={\left(P_{i1},P_{i2},\cdots ,P_{1D}\right)}^{T}$. At the same time, another important attribute of the population is the group extremum. The group extremum position vector is $P_{g}={\left(P_{g1},P_{g2},\cdots ,P_{gD}\right)}^{T}$.

Each particle updates its speed and position through the following iteration formula:

(2) $$V_{id}^{k+1}=\omega V_{id}^{k}+c_{1}r_{1}(P_{id}^{k}-X_{id}^{k})+c_{2}r_{2}(P_{gd}^{k}-X_{id}^{k})$$

(3) $$X_{id}^{k+1}=X_{id}^{k}+V_{id}^{k+1}$$

where ω is the inertia weight; d=1,2,⋯,D; i=1,2,…,n; k is the number of current iterations; $V_{id}$ is the speed of the particle; $c_{1}$ and $c_{2}$ are nonnegative constants, also called acceleration factors; and $r_{1}$ and $r_{2}$ are random numbers in the (0,1) interval. Particles are usually restricted to a certain range, where $X_{id}^{k}\in [L_{d},U_{d}]$ and $L_{d},U_{d}$ are the lower and upper limits of the search space, respectively, and $V_{id}^{k}\in [V_{\min,d},V_{\max,d}]$ and $V_{\min,d},V_{\max,d}$ are the minimum and maximum velocities, respectively. In general, these velocities are defined as $V_{\min,d}=-V_{\max,d}$, where 1≤d≤D,1≤i≤n.

The PSO algorithm has formed specific theoretical and experimental systems, which have been verified by many scientists. Thus, the PSO algorithm has a flowchart and steps. With in-depth research, its specific flowchart has been widely recognized and investigated. The specific steps of the process of the standard PSO algorithm [23] are as follows:

Initialize the particle population, and customize the initial speed and position of the particles.

(1) According to the fitness function, determine the fitness function value of each particle initialized.
(2) Compare the fitness value of each particle in the old position with that in the new position to determine the best position of the particle with high fitness. If the value of the particle in the new position is better, then the optimal position, $P_{id}$, of the particle, *i*, is replaced by the new location. If the fitness value of the new position is not as good as the fitness value of the original position, then retain $P_{id}$ as the optimal position.
(3) Compare the fitness value of each particle with that of other particles to determine the best position of particles with high fitness. If the new position is good, that is, the fitness value of the new particle is optimal, then the position of the new particle becomes $P_{gd}$. If the fitness value of the particle in the original global optimal position, $P_{gd}$, is better, then $P_{gd}$ remains as the global optimal position.
(4) According to Formulas (1) and (2), update the speed and position of each particle.
(5) If the termination condition is not satisfied, then return to Step 2. Otherwise, terminate this algorithm.

**Figure 1:**
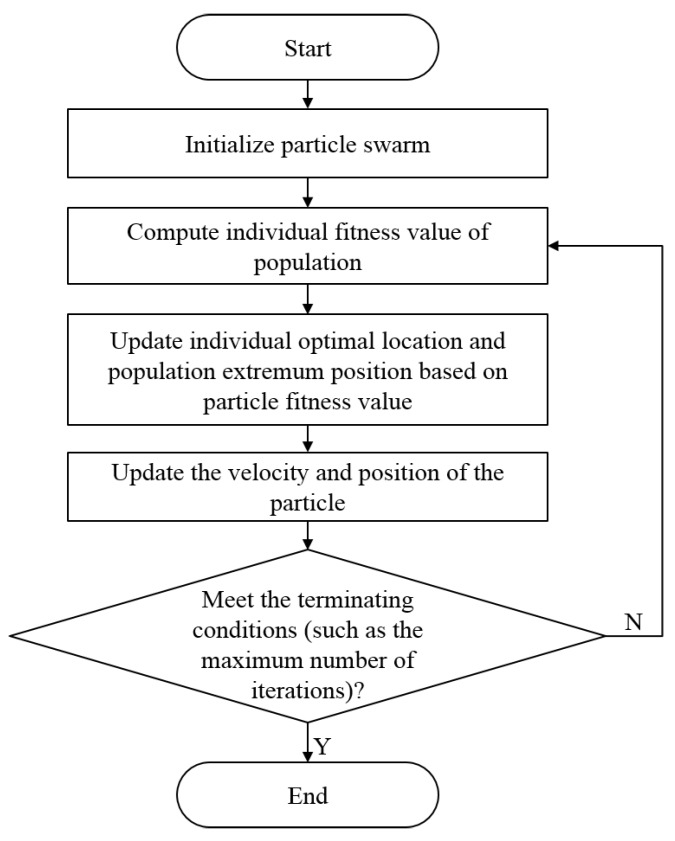
Flowchart of the standard PSO algorithm.

The flowchart of the specific standard PSO algorithm is shown in Figure 1.

### 2.3. Game Theory

GT proposes that two or more people change their countermeasures according to the tactics chosen by other people in an equal game and ultimately achieve the goal of winning or maximizing their interests [24,25]. A basic game model consists of three basic elements, namely, the participants, strategy sets, and utility functions. The definitions of these three basic elements are as follows:

(1). Participants. The participants, also known as the board member, are the decision-makers in the game. The participants choose their actions reasonably in the game process to maximize their interests. P={1,2,3,⋅⋅⋅,n} is set to represent *n* decision-makers involved in the game.
(2). Strategy sets. The strategy set stipulates the action plan for participants to respond to other participants. $s_{p}$ is used to represent a specific strategy for the participant, p. $S_{p}=\left\{s_{p}\right\}$ is utilized to indicate all possible strategies for the participant, p. Then, n participants choose one strategy to form the n-dimensional vector, $S=\left\{s_{1},s_{2},s_{3},\cdots ,s_{n}\right\}$. The vector is a strategy combination, where $s_{p}$ represents the strategy selected by the participant, *p*.
(3). Utility functions. The utility function is the benefit obtained by the participant after the game under a particular strategy combination, S, which can provide a more rational decision basis for the participants to continue to participate in the game. $u_{p}$ is used to represent the utility function of the participant, *p*. Under the influence of *n* participants’ actions, the final income of the participant, *p*, is $u_{p}=u_{p}\left(s_{1},s_{2},s_{3},\cdots ,s_{n}\right)$. $U=\left\{u_{1},u_{2},u_{3},\cdots ,u_{n}\right\}$ is used to represent the income of *n* participants in one game.

The ultimate goal of GT is to achieve a balanced state of interest by letting many people start a game. In the process of hyperspectral image dimension reduction, information entropy and interclass separability are the commonly used index evaluation criteria for measuring band selection. These two evaluation criteria should be optimized to achieve the best effect of dimension reduction. However, in fact, these two evaluation criteria cannot reach the optimum at the same time. Thus, we need to balance these evaluation criteria through GT. In this work, the dimension reduction method combining multiple evaluation criteria is considered a multi-objective optimization problem, and a conflict of interest exists in the process of multi-objective optimization. Therefore, this work introduces GT to coordinate the interest relationship between multiple targets, achieve the equilibrium state, and identify the band combination that ensures the best possible information entropy and interclass separability.

### 2.4. Evaluation Criterion Function

In this work, information entropy and *B* distance are selected as evaluation criteria.

(1) Information Entropy Between Bands

According to Shannon theory [26], entropy can be used to represent the information quantity and reflect the abundance of average information in images. For HSIs, assuming that their data are quantized as *L* bits, the entropy value of the *i* band images is derived as follows:

(4) $$E(i)=-\sum_{i=0}^{2^{L}-1}P_{i}(r)\log_{2}P_{i}(r)$$

where E(i) represents the entropy of the *i* band, *i* = 1, 2, …, *l*, *l* is the total number of bands in the image, *r* represents the gray value of the pixel, and $P_{i}(r)$ indicates the probability that the gray value of the *i* band is *r*. The greater the entropy is, the more the information. By calculating the entropy value of hyperspectral images, we can select the band combination with the greatest information entropy.

(2) *B* Distance between Classes

The *B* distance [27] represents the separability between two categories. The greater the *B* distance is, the stronger the separability between two categories. The *B* distance can consider the two statistical variables at the same time and is expressed as follows:

(5) $$B_{ij}=\frac{1}{8}{\left(\mu_{i}-\mu_{j}\right)}^{T}{\left(\frac{\sum_{i}+\sum_{j}}{2}\right)}^{-1}(\mu_{i}-\mu_{j})+\frac{1}{2}\ln\left[\frac{\left|\frac{\sum_{i}+\sum_{j}}{2}\right|}{{\left(\left|\sum_{i}\right|\left|\sum_{j}\right|\right)}^{\frac{1}{2}}}\right]$$

where $\mu_{i}$ and $\mu_{j}$ are the spectral mean of the two corresponding regions in the specified samples and ∑*_i_* and ∑*_j_* are the spectral covariance of the corresponding regions.

### 2.5. Dimension Reduction Steps

From the perspective of combining multiple evaluation criteria, this work considers dimension reduction as a multi-objective optimization problem because conflicts exist in the optimization process of multiple targets and optimizing multiple targets at the same time is difficult. GT is introduced to coordinate the relationship between multiple targets to make the goals as optimal as possible. The specific algorithm steps are as follows:

(1) Subspace Division

Some of the most important characteristics of hyperspectral image data are the large number of spectral bands and the high correlation and redundancy between adjacent bands. If the selection bands from all bands are bound to lose some of the key local characteristics, then the selected band combination may not help in improving the classification accuracy. The basic idea of solving these problems is to divide all bands into several subspaces and conduct band selection. Many subspace partitioning methods have been commonly used. This work adopts the adaptive subspace decomposition (ASD) method based on correlation filtering. This method was proposed by Zhang Junping et al. [28]. The method calculates the correlation coefficient, $R_{i,j}$, between two bands, where *i* and *j* denote the *i* and *j* bands, respectively. The range of $R_{i,j}$ is $-1\leq R_{i,j}\leq 1$. The greater the absolute value of the correlation coefficient is, the stronger the correlation between bands. The closer the value is to 0, the weaker the correlation. The definition of $R_{i,j}$ is expressed as follows:

(6) $$R_{i,j}=\frac{E\left\{\left(x_{i}-\mu_{i})(x_{j}-\mu_{j}\right)\right\}}{\sqrt{E\left\{{\left(x_{i}-\mu_{i}\right)}^{2}\right\}}\sqrt{E\left\{{\left(x_{j}-\mu_{j}\right)}^{2}\right\}}}$$

where $\mu_{i}$ and $\mu_{j}$ are the mean values of $x_{i}$ and $x_{j}$, respectively, and E(⋅) represents the mathematical expectation for the functions in brackets. According to the correlation coefficient matrix, R, obtained using Formula (5), the corresponding threshold, *T*, is set, and the continuous wave band satisfying $R_{i,j}\geq T$ is combined into a new subspace. By adjusting *T*, the number of bands and the number of subspaces in each subspace can be adaptively changed. With the increase in *T*, the number of bands in each space decreases and the number of subspaces increases. The correlation between the spectral bands of hyperspectral images has a partitioned feature, and the correlation coefficient matrix, *R*, can quantitatively reflect this block feature, thus dividing the strong continuous band in a subspace according to the feature of the block. Then, bands are selected in each subspace to form a band combination to reduce the correlation between bands.

(2) Initialization of Particle Swarm

Each particle consists of a hyperspectral image band combination, inertia weight, ω, and acceleration factor, C. In the initial population, the three parts of the particles mentioned previously are randomly generated, and the initial velocity and location of the particles are defined. The initial population size is reasonably set according to the complexity of the calculation to ensure that the initial population contains more possible solutions. The total number of individuals in the population is assumed to be *m*, and *N* subspace is divided under the subspace division method in Step (1). Then, the *M* bands are randomly selected in each subspace, and the band combination of *N* × *M* bands is used as the individual in the population. In this manner, *m* individuals can be initialized randomly under the constraint of subspace partition.

(3) Establishing an Elite Solution Set

The fitness values of the particles in the population are compared, the eligible particles are detected, and the qualified particles are placed in the non-inferior solution set. In solving multi-objective optimization problems, the most important thing is to solve non-inferior solutions and non-inferior solution sets. The so-called non-inferiority solution is the existence of a problem solution in the feasible domain of the multi-objective optimization problem, so that other feasible solutions are not inferior to the solution. Then, the solution is called a non-inferior solution, and the set of all non-inferior solutions is a non-inferior solution set. In the process of reducing the dimension, the band combination with large information entropy and a large *B* distance between the selected classes, a and b, at the same time is a non-inferior solution and is placed in the non-inferior solution set. The solution in a non-inferior solution set is the optimal location of the individual history. In the zeroth generation, a global optimum is selected from the individual historical optimal location.

An elite solution set is set up. The elite solution set is initialized as empty sets, and at the beginning of the algorithm, a solution extracted from the zeroth generation is stored in the elite solution set. The non-inferiority hierarchy of the new species group is calculated by the non-dominated solution sorting method presented in the literature [29]. The band combination that is non-inferior and a high level is the elite solution band combination and is added to the elite solution concentration. The elite solution set is used to preserve the band combination of these elite solutions. Then, in the process of algorithm iteration, the band combination of these elite solutions is added to the algorithm population to participate in an iterative search. The competition of the elite band combination and the algorithm population in the subsequent iteration to produce the next-generation population and maintain the excellent population is advantageous.

(4) Game Decision Making

In the literature [30], a clonal selection algorithm is proposed to solve preference multi-objective optimization. Inspired by this algorithm, this work constructs a finite repeated game model based on goal preference. The information entropy and *B* distance are regarded as the participants of the game. The two participants perform a limited repeated game in the iterative process of the algorithm. Given that the game repeats many times, the rational participants need to weigh the immediate and long-term interests when they select the strategic action at the current game stage. The participants may give up their immediate interests for achieving the long-term interests. Then, the strategy that can maximize the profits may not be selected in the current game. Therefore, in the game process, cooperation and confrontation exist among the participants. According to the earnings of the previous stage, deciding whether to select cooperation or confrontation in the subsequent stage of the game is possible. The specific game model is as follows:

The fitness values of the entire population in information entropy and the *B* distance are expressed as $\left(E_{1}\left(t\right),E_{2}\left(t\right),\cdots ,E_{m}\left(t\right)\right)$ and $\left(B_{1}\left(t\right),B_{2}\left(t\right),\cdots ,B_{m}\left(t\right)\right)$, respectively. In the subsequent iteration of *T*, the fitness matrix, FITS(t), can be expressed as follows:

(7) $$FITS\left(t\right)=\left[\begin{array}{l} E_{1}(t),E_{2}(t),\cdots ,E_{m}(t)\\ B_{1}(t),B_{2}(t),\cdots ,B_{m}(t) \end{array}\right]$$

where *m* is the total number of individuals in the population. Then, a utility matrix is established and is used to represent the income of each participant in the strategy. The utility matrix, U(t), is expressed as follows:

(8) $$U\left(t\right)=\left[\begin{array}{l} u_{11}(t),u_{12}(t)\\ u_{21}(t),u_{22}(t) \end{array}\right]$$

where $u_{pq}\left(t\right)$ represents the income value brought by participant *p* to participant *q*. The ultimate goal of each participant is to obtain maximum benefits. The maximized benefit in dimension reduction is to determine the maximum entropy or *B* distance in the band combination.

At the beginning of the game, the participants attempt to make some strategic actions and expect their own and the game’s final strategic actions to make their own gains as much as possible. If the strategic action made in the previous stage makes the income increase, then the follow-up stage selects the reward strategy as much as possible in the strategic action choice. Otherwise, the penalty strategy is selected. Therefore, the policy set, $S=\left\{s_{1},s_{2}\right\}$, is defined. Two participants share a strategy set, where $s_{1}$ indicates reward and $s_{2}$ indicates punishment. A reward selection probability matrix, PRI(t), is set up as follows:

(9) $$PRI\left(t\right)=\left[\begin{array}{l} P_{11}(t),P_{12}(t)\\ P_{21}(t),P_{22}(t) \end{array}\right]$$

where $P_{pq}\left(t\right)$ indicates that the probability value of the reward selection strategy for participant *q* is selected by participant *p*. Accordingly, the probability value of the punishment strategy is $1-P_{pq}\left(t\right)$. The participant, *q*, can adjust the reward selection probability matrix value of participant *p* to $P_{pq}\left(t\right)$ according to his actual income. If $u_{pq}(t)-\frac{1}{2}\sum_{p=1}^{2}u_{pq}(t)>0$ indicates that the strategic action taken by participant *p* eventually increases the benefit obtained by participant *q*, then participant *q* adjusts the PRI(t) matrix and increases the reward selection probability, $P_{pq}\left(t\right)$, for participant *p*. Conversely, participant *q* reduces $P_{pq}\left(t\right)$.

Based on the previously presented definition, a weight matrix, W(t), of the preference degree is established to reflect the strategic actions taken by the participants and expressed as follows:

(10) $$W\left(t\right)=\left[\begin{array}{l} w_{11}(t),w_{12}(t)\\ w_{21}(t),w_{22}(t) \end{array}\right]$$

where $w_{pq}\left(t\right)$ indicates the degree of preference of participant *p* to participant *q*. If participant *p* selects the reward strategy for participant *q*, then the value of $w_{pq}\left(t\right)$ increases; otherwise, it decreases. Given that the information entropy and *B* distance may be different, the normalization method is used to return the fitness values of each target to [0,1] to prevent the small numerical information from being flooded by large numerical information. Therefore, the mapping fitness function value of the multi-objective fitness function value of individual *k* on participant *i* is expressed as $F_{k}^{i}(t)$, as follows:

(11) $$F_{k}^{i}(t)=w_{i1}{E^{\prime }}_{k}(t)+w_{i2}{B^{\prime }}_{k}(t)$$

where ${E^{\prime }}_{k}(t)$ denotes the normalized information entropy and ${B^{\prime }}_{k}(t)$ denotes the normalized *B* distance. After a game is completed by two participants, the subsequent iteration process of the multi-objective PSO is performed with the mapping fitness function values of the two participants.

(5) Iteration of Multi-Objective PSO

According to Formula (1), the velocity of the particles in a population is calculated. The velocity of the particles is affected by the global and individual optimum particles. Among them, the global optimum particle is a randomly selected particle from the non-inferior solution set. After the particle velocity is updated, the new speed of the particle is observed to exceed the speed limit. Thus, the speed changes according to Formula (1). According to Formula (2), the position of the particles in the population is updated, so that the location of the new particles exceeds the location band. In the updating process, we determine whether the new position exceeds the range of the band group in which it is located, for example, the velocity of a particle is moved to the boundary. Moreover, the velocity and direction of the particle are rearranged, the velocity direction of the particle is changed, and the velocity of the particle is reduced to make the particle move in the opposite direction. During the line search, the individual historical optimal location and group historical location of the population are updated. The updating process can be divided into two steps. First, the new non-inferior solution set is combined with the old non-inferior solution set. Then, the new non-inferior solutions are removed from the newly generated non-inferior solution set, and the new set of non-inferior solutions is selected. The non-inferiority level of the set is computed and added to the elite solution set if it is an elitist solution.

(6) Check the End Condition

**Figure 2:**
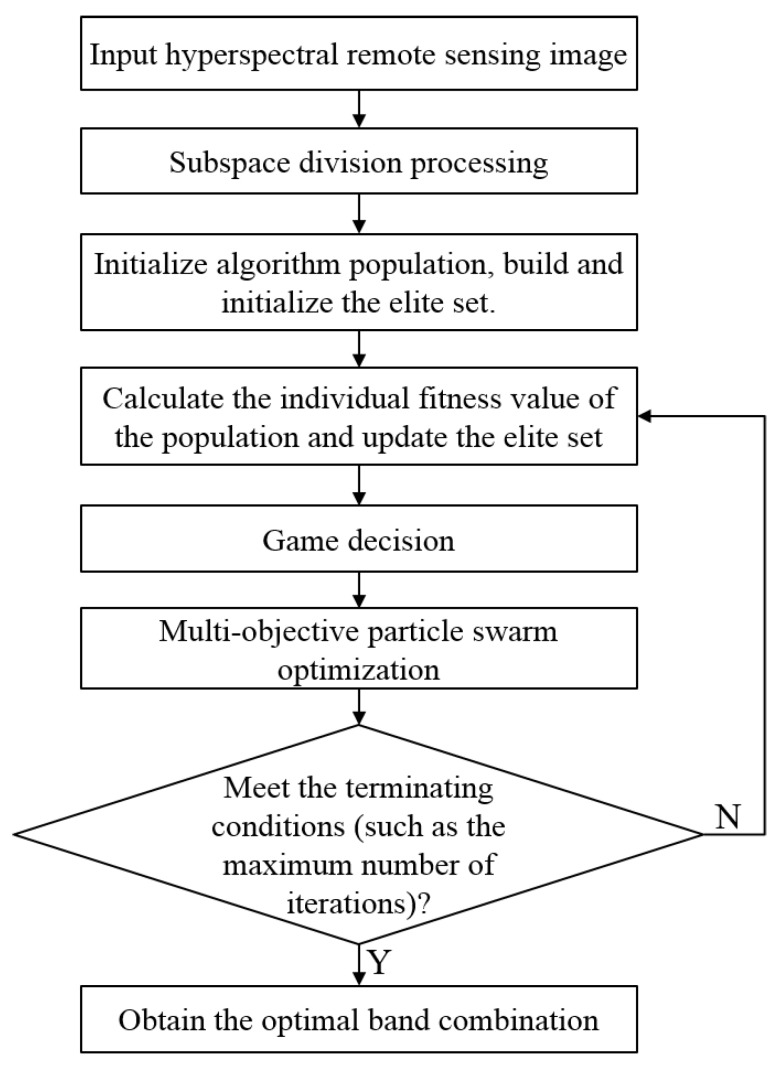
Flowchart of the proposed dimension reduction method.

If the check satisfies the termination condition (such as the maximum number of iterations), then it satisfies, terminates, and outputs the non-dominated band combination solution in the external set. The process of the proposed hyperspectral image dimension reduction method based on multi-objective PSO and GT is shown in Figure 2.

## 3. Experiments and Result Analysis

This work selects two hyperspectral images to verify the effectiveness of the proposed method. The experimental program is implemented by MATLAB (R2009b), and the support vector machine (SVM) classifier is implemented by the LIBSVM toolbox developed by Professor Lin Zhiren (Lin Chih-Jen) of the National Taiwan University. (http://www.csie.ntu.edu.tw/~cjlin/libsvm/). In this section, the details of the experimental data will be introduced first. Then, the subspace decomposition and experimental results will be given.

### 3.1. Experimental Data

**Figure 3:**
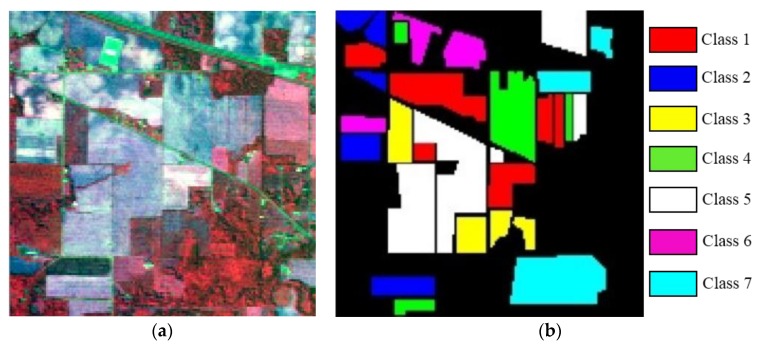
The AVIRIS dataset: (**a**) False color image synthesized by band 89, 5, and 120; (**b**) label map.

The first image is captured by the AVIRIS (Airborne Visible Infrared Imaging Spectrometer) sensor in the remote sensing experimentation area in Northwest Indiana, USA in June 1992. The AVIRIS sensor was flown for the first time in 1986 (first airborne images), first science data in 1987. Its data can provide a spatial resolution of 20 m and 224 spectral bands, covering a spectral range of 0.4 μm–2.5 μm, with a spectral resolution of 10 nm. The polluted bands, such as water vapor noise (bands 1–4, 78, 80–86, 103–110, 149–165, 217–224), are removed from the original bands, and the remaining 179 bands are used for the experiments. Figure 3 displays the false color image synthesized by band 89, 5, and 120 and the label map of the ground truth. Seven kinds of ground truth were used for the classification experiment. In each class, there are 25% of the samples used for the training set and 75% used for the test set. Table 1 shows the details of the AVIPIS dataset.

**Figure 4:**
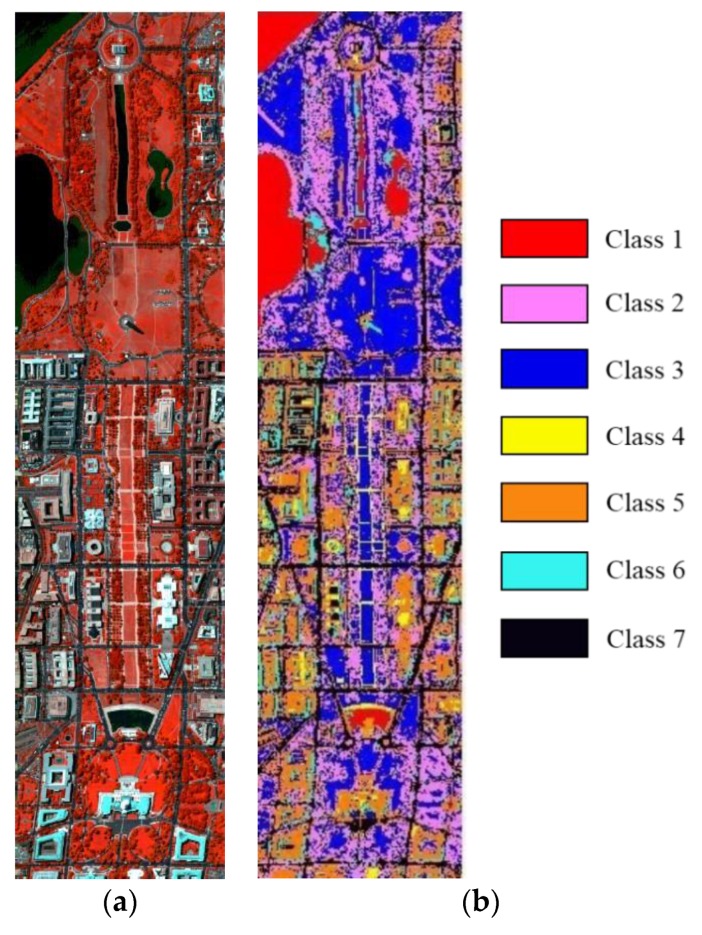
The HYDICE dataset: (**a**) False color image synthesized by band 63, 27, and 17; (**b**) label map.

The second image is captured by HYDICE (Hyperspectral digital imagery collection experiment) in Washington DC Mall. It contains 307 × 1280 pixels and 191 spectral bands in the wavelength range 400–2400 nm. Figure 4 displays the false color image synthesized by band 63, 27, and 17 and the ground truth of this data. The spatial resolution of HYDICE is high and can reach about 1 m. Therefore, some objects can be distinguished by visual measurement from Figure 4a. This dataset has seven classes. Table 2 shows the number of train samples and test samples in the HYDICE dataset. As can be seen, the ratio of the number of training samples to the number of test samples is 1:3.

### 3.2. Subspace Decomposition

**Figure 5:**
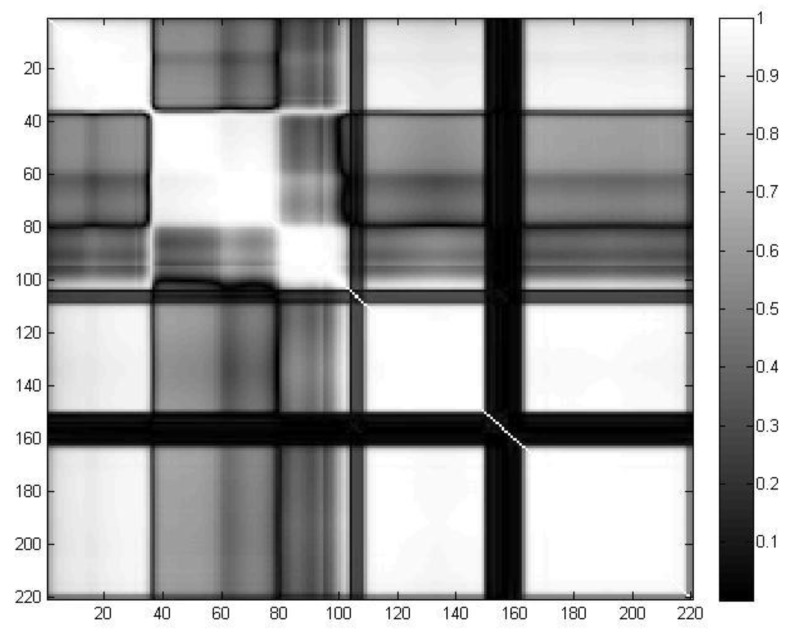
Grayscale diagram of the correlation coefficient matrix of the AVIRIS dataset.

The ASD based on relevance filtering is adopted. The correlation coefficients between various bands are obtained. The correlation coefficient matrix grayscale diagram of the AVIRIS dataset is shown in Figure 5 (the abscissa and ordinate denote the band numbers of the HSIs). In Figure 5, the brighter the point is, the higher the correlation coefficient. The brightest points represent a correlation coefficient of 1. The correlation coefficients of the points in the diagonal lines of the matrix are all 1. As can be seen from the correlation coefficient matrix, the hyperspectral image has a distinct block characteristic. Therefore, subspace decomposition based on the correlation between bands can reasonably divide the bands into groups. The subsequent processing in the subspace can also effectively improve the processing speed of hyperspectral data. Thus, the efficiency of dimension reduction and classification can be improved.

The number of subspaces is set to 5 to make the grouping characteristics of the hyperspectral images more obvious. The band sets contained in each subspace are shown in Table 3.

### 3.3. Classification Accuracy Evaluation

In this work, an error matrix is used for the classification accuracy evaluation. Table 4 shows a typical error matrix, which is used to evaluate the classification accuracy of *k* classes. The error matrix is a square array of *k* × *k*. Assuming that $x_{i,j}$ is the number of observation points in column j of line i, $x_{{}_{i+}}$ and $x_{+j}$ denote the sum of row i and column j, respectively, and *N* is the total number of test samples.

The main diagonal element, $x_{i,i}$, of the matrix indicates the number of pixels that are classified correctly. The elements outside the diagonal line are the number of misclassified samples. According to the error matrix, the evaluation index used to measure the classification accuracy can be obtained. These indexes include the producer’s accuracy (PA), user’s accuracy (UA), and overall accuracy (OA).

The PA refers to the ratio of the number of pixels correctly classified into a certain class to the total number of actual pixels that belong to that class. It can be expressed as follows:

(12) $$PA_{i}=\frac{x_{i,j}}{x_{+i}}$$

The UA refers to the ratio of the number of pixels correctly classified into a certain class to the total of pixels classified into that class. It can be expressed as follows:

(13) $$UA_{i}=\frac{x_{i,j}}{x_{i+}}$$

The OA refers to the ratio of the number of pixels correctly classified to the total number of all labeled pixels. It can be derived as follows:

(14) $$OA=\frac{\sum_{i=1}^{k}x_{i,i}}{N}$$

### 3.4. Dimension Reduction and Classification Experiment

The proposed method considers three basic criteria, namely, the inter-band correlation, information entropy, and interclass separability. Four groups of comparative experiments, namely, groups A, B, C, and D, are designed to verify the advantage of the proposed method. Group A uses the dimension reduction method in [31], where information entropy is adopted as the evaluation criterion. Group B adopts the dimension reduction method using the Bhattacharyya distance as the evaluation criterion [24]. Group C transforms the information entropy and interclass separability into a single evaluation criterion by weighting. Group D adopts the proposed method. The SVM [13] classifier is used for classification to verify the effect of dimension reduction. The parameters of the multi-objective PSO algorithm in this work are set as follows: The particle dimension is 5, the population number is 50, the acceleration factors, C1 and C2, are 0.8, and the maximum iteration number is 1000. The inertia weight ranges from 0.1 to 1.2, which changes in accordance with Formula (15):

(15) $$\omega =\omega_{\max}-\frac{\omega_{\max}-\omega_{\min}}{i_{\max}}\times i$$

where i is the number of current iterations and $i_{\max}$ is the maximum number of iterations. Each particle represents a band combination. The particles in the initial population are randomly generated from the band combination, and the initial velocity of the particles is 0.

The parameters of the SVM classifier are set as follows: The SVM selects the RBF (Radial Basis Function) kernel function. The penalty parameter, *c*, and kernel parameter, *γ*, are selected. After half-off cross-validation, *c* = 16 and *γ* = 2.2974.

The multi-objective optimization problem usually has more than one optimal solution sets. Therefore, from the experimental results of four groups, five optimal band combinations are extracted and compared. Table 5, Table 6, Table 7 and Table 8 lists the details of the experimental results of each group.

As can be seen from Table 5, the accuracy obtained by the first band combination is obviously higher than that obtained by the remaining band combination. It should be noted that all the band combinations in Table 5 are optimal band combinations in Group A. In other words, only one band combination is quite close to the global optimum solution. Additionally, the remaining band combination only achieves the local optimum. Similarly, the same conclusion can be drawn from Table 6 and Table 7. Therefore, these methods used in Group A, B, and C easily fall into the local optimum. However, the accuracies shown in Table 8 are quite close each other and four accuracies exceed 81%, which are very close to the global optimum. Overall, the accuracies in Table 8 are higher than those in Table 5, Table 6 and Table 8. To sum up, the proposed method more easily achieves the global optimum compared to both the method based on a single evaluation criterion and the method based on multiple evaluation criterions, but without combing GT. Furthermore, the proposed method provides better adaptability and can provide higher accuracy for hyperspectral sensor data classification.

**Figure 6:**
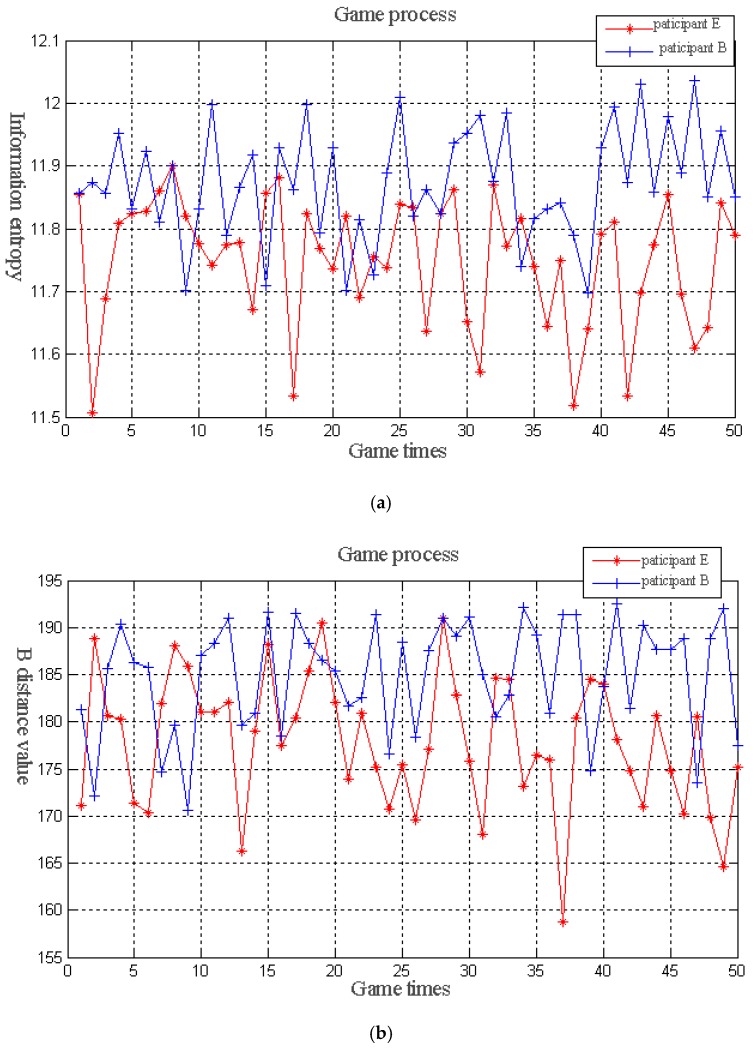
(**a**) Information entropy in the game process. (**b**) *B* distance in the game process.

Figure 6 shows the game process of group D. Figure 6a shows the changes of the information entropy of the optimal individual. Figure 6b shows the changes of the *B* distance of the optimal individual. Taking Figure 6a as an example, in the early stage of the game, participant E (information entropy) observed that the information entropy of the optimal individual detected by participant B (*B* distance) is often slightly larger than that found by itself. This finding indicates that participant B can help participant E in identifying the individual with more information entropy. Therefore, in the subsequent game, participant E’s preference for participant B is enhanced; that is, reward strategies are chosen to ensure that participant B identifies the individuals with better information entropy. The corresponding probability value, $P_{pq}\left(t\right)$, of the selection reward strategy in the reward selection probability matrix, PRI(t), increases. Thus, the value of the preference degree, $w_{pq}\left(t\right)$, in the weight matrix, W(t), increases. By contrast, when participant E determines that the information entropy of the optimal individual detected by participant B is often slightly smaller than that found by itself during the game, and under the condition of maintaining the original preference, the entropy value of the optimal individual identified by participant E is reduced by the shadow of participant B. This finding indicates that participant B at this time cannot help participant E identify the individual with better entropy, but it can “hinder” participant E in finding the optimal individual. Then, in the subsequent game, participant E weakens the preference for participant B; that is, by reducing the corresponding probability value, $P_{pq}\left(t\right)$, of the selection reward strategy in the reward selection probability matrix, PRI(t), under the reward strategy. Thus, the value of the preference degree, $w_{pq}\left(t\right)$, in the weight matrix, W(t), is decreased to reduce or weaken the “hindrance” to finding the optimal individual, which is caused by participant B. Then, individuals with good information entropy can be possibly identified under the existing conditions. In addition, participant B requires an individual that can make the *B* distance more valuable. At this time, the two participants assume that the strategies they adopted are optimal, thus achieving a balanced state.

**Figure 7:**
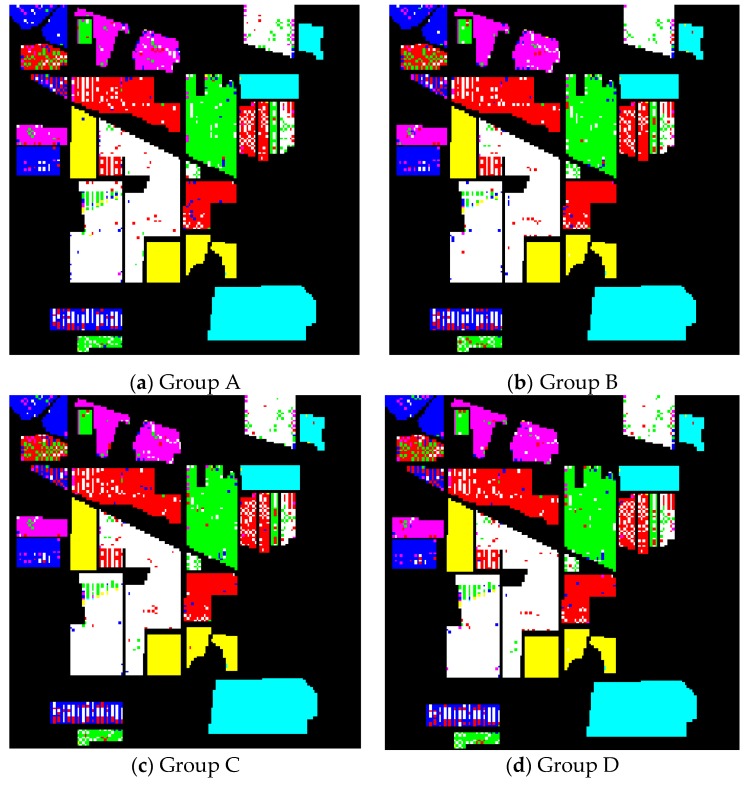
Classification maps of four groups for the AVIRIS dataset.

Figure 7 displays the classification results corresponding to the band combination with the highest classification accuracy in groups A, B, C, and D. The error points shown in Figure 7a,b are more than those shown in Figure 7c,d. This finding indicates that the classification effect under a single evaluation criterion is not as good as that under comprehensive multiple evaluation criteria. Through careful observation, we determine that the error points in (c) are slightly more than those in (d), and the classification accuracy of (c) is slightly worse, which demonstrates the effectiveness and superiority of the proposed method.

The experiments show that, compared with the band combination obtained by a single criterion or multiple criteria without a game, the band combination obtained by the proposed method is better, because the proposed method adopts the GT to coordinate the two evaluation criteria, that is, information entropy and interclass separability. This work proposes a hyperspectral image dimension reduction method based on PSO and GT (PSO-GT). To further evaluate the performance of the proposed method, the proposed method is compared with several methods, which combine existing intelligent algorithms with GT. These methods include the method based on GA and GT (GA-GT), the method based on simulated annealing GA and GT (SAGA-GT), and the method based on a differential evolution algorithm and GT (DE-GT). The experimental results on two datasets under the best band combination are shown in Table 9 and Table 10, respectively.

As shown in Table 9, the PA and UA in the PSO-GT experiments are generally better than those in the other experiments, which indicates that the omission error and commission error generated by the proposed method are smaller. The OA of SAGA-GT is only 0.54% higher than that of GA-GT. However, from the PA and UA of each class, the method based on SAGA-GT is better than the method based on GA-GT. The OA, PA, and UA of SAGA-GT and DE-GT are close, and all of them are lower than those of PSO-GT. Similarly, Table 10 demonstrates that the proposed method is equipped with better performance than the comparison methods. In summary, the method proposed in this work is an effective hyperspectral image dimension reduction method.

To better evaluate the performance of the proposed method, the works of Li et al. [27], Xu et al. [32], and Shen et al. [33] are compared with the proposed method. In [31], a band selection method based on a particle swarm dynamic with sub-swarm optimization was proposed for dimension reduction of hyperspectral sensor data. According to the features of multiple bands and the strong mutual correlation among these bands, the work of [32] improved the PSO algorithm for band selection of hyperspectral sensor data. Table 11 displays the OA obtained by the comparison methods, and the division of the training set and test set is the same as Table 1 and Table 2. As can be seen from Table 11, the performance of the proposed method obviously outperforms all comparison methods.

## 4. Conclusions

In this work, the correlation between bands was adopted as a constraint, both information entropy and interclass separability were adopted as evaluation criteria, and the dimension reduction of hyperspectral images was considered as a multi-objective optimization problem. In this way, PSO was used to search for the best band combination that can result in optimal multiple objectives, and GT was applied to coordinate the interests of all objectives. The experimental results demonstrated that the proposed method can search for the better band combination that is more conducive to improving classification accuracy than that searched by the dimension reduction method based on either interclass separability or information entropy and the method based on multiple criteria, but without combining GT. The dimension reduction method proposed in this work still has room for further optimization. A possible limitation of the proposed method is the large computational cost caused by too much evaluation criterion. Better results could be obtained if more evaluation criterions were considered. However, a greater number of evaluation criterions also increases the computational cost of the game process. Furthermore, in the game model, the probability change range of the choice reward strategy and the preference weight value will affect the search for the optimal band combination. Therefore, the game model will be improved in this regard.

## Figures and Tables

**Table 1:** The number of train samples and test samples in the AVIRIS dataset.

Class	Name	Train	Test
c1	Corn-notill	239	717
c2	Corn-mintill	139	417
c3	Grass-trees	124	373
c4	Soybean-notill	161	484
c5	Soybean-mintill	411	1234
c6	Soybean-clean	102	307
c7	Woods	216	647
Total		1392	4179

**Table 2:** The number of train samples and test samples in the HYDICE dataset.

Class	Name	Train	Test
c1	Water	306	918
c2	Trees	101	304
c3	Grass	482	1446
c4	Path	44	131
c5	Roofs	959	2875
c6	Street	104	312
c7	Shadow	24	73
Total		2020	6059

**Table 3:** Subspace decomposition dimension and its band.

Subspace	1	2	3	4	5
bands	5–35	36–76	77,79,87–97	98–102	111–148,166–216
band number	31	41	13	5	89

**Table 4:** Error matrix.

	Ground Reference Information						Total per Row
Classification results	Class	1	2	3	…	k	
	1	$x_{1,1}$	$x_{1,2}$	$x_{1,3}$	…	$x_{1,k}$	$x_{1+}$
	2	$x_{2,1}$	$x_{2,2}$	$x_{2,3}$	…	$x_{2,k}$	$x_{2+}$
	3	$x_{3,1}$	$x_{3,2}$	$x_{3,3}$	…	$x_{3,k}$	$x_{3+}$
	⋮	⋮	⋮	⋮	⋮	⋮	⋮
	k	$x_{k,1}$	$x_{k,2}$	$x_{k,3}$	…	$x_{k,k}$	$x_{k+}$
Total per column		$x_{+1}$	$x_{+2}$	$x_{+3}$	…	$x_{+k}$	N

**Table 5:** Experimental results of group A.

Group	Band Combination	Information Entropy	Accuracy
A	(25,37,42,89,133)	12.0544	80.1292
	(25,37,38,88,120)	12.0523	77.7605
	(27,37,41,90,124)	12.0385	78.2630
	(24,37,38,90,125)	12.0363	77.8323
	(21,37,38,88,123)	12.0192	78.6697

**Table 6:** Experimental results of group B.

Group	Band Combination	B Distance	Accuracy
B	(29,37,72,98,137)	194.4588	80.9068
	(28,37,69,99,139)	193.7285	80.8948
	(30,37,72,98,141)	193.0367	80.7991
	(29,37,69,98,134)	192.8221	81.2657
	(30,37,73,96,138)	192.7607	80.4163

**Table 7:** Experimental results of group C.

Group	Band Combination	Information Entropy	B Distance	Accuracy
C	(29,37,72,97,139)	11.8069	194.6254	80.7632
	(30,37,55,97,136)	11.7785	194.2067	80.7034
	(29,37,74,99,137)	11.7858	194.0661	80.9547
	(28,37,70,96,136)	11.7269	193.3245	81.1461
	(27,37,57,98,140)	11.8559	193.0160	80.5838

**Table 8:** Experimental results of group D.

Group	Band Combination	Information Entropy	B Distance	Accuracy
D	(25,37,65,96,135)	11.7025	185.9396	81.2537
	(30,37,73,98,132)	11.7740	191.0403	81.1341
	(20,37,70,98,123)	11.6766	184.1740	81.0145
	(31,37,71,97,136)	11.8158	192.1826	81.0025
	(30,37,52,97,133)	11.8732	188.7685	80.7513

**Table 9:** Results of classification experiments for the AVIRIS dataset.

Method	GA-GT		SAGA-GT		DE-GT		PSO-GT	
Class	PA	UA	PA	UA	PA	UA	PA	UA
c1	0.8082	0.3787	0.7941	0.3886	0.7933	0.3874	0.7907	0.7907
c2	0.8229	0.8047	0.8035	0.8316	0.8054	0.8126	0.8008	0.8145
c3	0.8141	0.8412	0.7951	0.8252	0.8034	0.8097	0.8573	0.8344
c4	0.8218	0.5724	0.8260	0.6493	0.8246	0.6225	0.8104	0.7845
c5	0.7784	0.8713	0.8034	0.8543	0.8047	0.8413	0.8065	0.8001
c6	0.8054	0.8687	0.7957	0.8645	0.7998	0.8378	0.8089	0.8208
c7	0.8255	0.6041	0.8105	0.7492	0.8025	0.8486	0.8358	0.8391
OA	79.54%		80.08%		80.48%		81.25%	

**Table 10:** Results of classification experiments for the HYDICE dataset.

Method	GA-GT		SAGA-GT		DE-GT		PSO-GT	
Class	PA	UA	PA	UA	PA	UA	PA	UA
c1	0.7884	0.8213	0.8024	0.8543	0.8147	0.8413	0.8268	0.7968
c2	0.8054	0.8047	0.8027	0.8345	0.8078	0.8328	0.8187	0.8138
c3	0.8245	0.6648	0.8125	0.7692	0.8135	0.8474	0.8368	0.8248
c4	0.8278	0.8017	0.8125	0.8236	0.8132	0.8016	0.8135	0.8025
c5	0.8032	0.3587	0.7941	0.3896	0.7968	0.3984	0.8023	0.7912
c6	0.8148	0.5824	0.8236	0.6889	0.8328	0.6238	0.8136	0.7839
c7	0.8169	0.8312	0.8051	0.8152	0.8124	0.8037	0.8483	0.8214
OA	81.62%		82.05%		82.18%		83.46%	

**Table 11:** Comparison with other methods (OA).

Dataset	PSO-GT	Li et al. [27]	Xu et al. [32]	Shen et al. [33]
The AVIRIS dataset	81.25%	79.16%	80.36%	79.58%
The HYDICE dataset	83.46%	80.28%	81.79%	81.02%

## Data Availability

The data used to support the findings of this work are available from the corresponding author upon request.
